# Metabolic-related markers and inflammatory factors as predictors of dyslipidemia among urban Han Chinese adults

**DOI:** 10.1186/s12944-019-1109-1

**Published:** 2019-08-31

**Authors:** Ying Lian, Lingling Xie, Yafei Liu, Fang Tang

**Affiliations:** 1 Center for Data Science in Health and Medicine, Shandong Provincial Qianfoshan Hospital, The First Affiliated Hospital of Shandong First Medical University, Jingshi Road 16766, Jinan, 250014 China; 2 Shandong Provincial Qianfoshan Hospital Affiliated to Shandong University, Jinan, China; 3 Department of Endocrinology, Zhangqiu District Hospital of Traditional Chinese Medicine, Jinan, China

**Keywords:** Metabolic-related markers, Inflammation, Dyslipidemia, Mediation, Cohort study

## Abstract

**Background:**

Metabolic-related markers and inflammatory factors have been proved to be associated with increased risk of dyslipidemia. Elucidating the mechanisms underlying these associations might provide an important perspective for the prevention of dyslipidemia. In the present study, we aimed to explore the effect of metabolic-related markers on dyslipidemia, and to assess what extent inflammation mediating these associations.

**Methods:**

A total of 25,130 participants without dyslipidemia at baseline were included in the present study during 2010–2015. A partial least squares path model was used to explore possible pathways from metabolic-related markers to dyslipidemia, and the mediation role of inflammation.

**Results:**

Lipid metabolism factor, blood pressure factor, obesity condition factor, glucose metabolism factor, renal function factor and lifestyle factor had diverse impact on development of dyslipidemia, directly and (or) indirectly. Partial least squares path analysis revealed that the determination coefficient of the model (R^2^) was 0.52. Lipid metabolism factor, obesity condition factor, and glucose metabolism factor had both direct and indirect effect on dyslipidemia through inflammatory factor. Lipid metabolism factor was the most important risk factor (β = 0.68) in the prediction of dyslipidemia, followed by obesity condition factor (β = 0.06) and glucose metabolism factor (β = 0.03).

**Conclusions:**

Metabolic-related markers are strong risk factors for dyslipidemia. Inflammatory factors have significant mediating effect on these relationships. These findings suggested that comprehensive intervention strategies on metabolic biomarkers and inflammatory factors should be taken into consideration in prevention and treatment of dyslipidemia.

## Background

Dyslipidemia has become an emerging epidemic worldwide [1, 2]. Based on a report from American College of Cardiology, about 39% of the global population has elevated cholesterol, and more than one-half of those individuals live in developed countries [3]. Epidemiological studies conducted in China showed that the prevalence of dyslipidemia significantly increased in recent years [4, 5]. The Global Burden of Disease, Injuries, and Risk Factor study indicated that during the past decades worldwide morbidity and mortality attributable to dyslipidemia have increased by 26.9 and 28.0%, respectively [6].

Metabolic-related risk factors such as glucose level and hypertension have been proved to be associated with dyslipidemia [7, 8]. Most of these factors reflect different metabolic pathways that may be involved in the pathogenesis of dyslipidemia. However, previous studies mainly focused on estimating the associations between risk factors and dyslipidemia or improving the prediction of dyslipidemia using these biomarkers [9–12]. Few studies have attempted to evaluate a comprehensive panel of multiple metabolic-related factors representing distinct pathogenetic pathways for predicting the development of dyslipidemia. Elucidating the mechanisms underlying the associations between biomarkers and dyslipidemia by pathway analysis might provide a novel perspective for the research of dyslipidemia.

Inflammation is receiving increasingly attention for its potential role in the pathogenesis of metabolic disorders including dyslipidemia [13–15]. Individuals with dyslipidemia have higher levels of inflammatory biochemical markers than those without dyslipidemia [15]. Most of available studies implicating inflammation are based on measurements of pro-inflammation cytokines and chemokines, which are limited for laboratory methods, especially in a large sample of population. Routine check-up biomarkers such as white blood cell count (WBC), γ-glutamyltransferase (GGT) are well-recognized indicators of inflammation [16, 17]. Moreover, several studies have found the mediating role of systemic inflammation in the effect of metabolic-related factors on metabolic diseases such as hypertension and subclinical atherosclerosis [18, 19]. Therefore, we hypothesized that inflammation may play a mediation role in development of dyslipidemia, mediating at least part of the associations between metabolic-related markers and dyslipidemia.

In the present study, data from a perspective cohort of Chinese routine health check-up population were used to explore the effect of metabolic-related markers on dyslipidemia, and the mediation role of inflammatory factors among this relationship.

## Methods

### Study population

The large-scale perspective cohort study was set up in 2010 based on the routine health check-up system in the Center for Health Management of Shandong Provincial Qianfoshan Hospital. Participants free of dyslipidemia, diabetes, cardiovascular disease, hepatosis, renal dysfunction, and hypothyroidism at baseline were recruited, and followed up for dyslipidemia with well-designed clinical and laboratory examinations. The cohort was selected from participants who took the first health check in 2010, 2011, 2012, 2013 or 2014 respectively and at least took health check-up 2 times in the 5-years follow-up. A total of 25,130 participants were included in the analyses.

### Measurements of anthropometric and lifestyle variables

Anthropometric data were collected following a standardized procedure. Height and weight were measured while participants wore light clothes without shoes. The body mass index (BMI) was calculated as weight (kg) divided by height squared (m^2^). Smoking and drinking statuses were classified as “never”, “former”, or “current” according to self-reported questions.

### Measurements of biochemical biomarkers

Venous blood samples were drawn after at least 12 h of fasting for laboratory tests. Systolic blood pressure (SBP) and diastolic blood pressure (DBP) were measured on the right arm of seated participants by a physician using Omron HEM-907 (Quick Medical) by the cuff-oscillometric method. Blood biochemical biomarkers including white blood cell count (WBC), neutrophile granulocyte (NGC), monocyte count (MCC), triglyceride (TG), high-density lipoprotein cholesterol (HDL-C), total cholesterol (TC), low-density lipoprotein cholesterol (LDL-C), γ-glutamyltransferase (GGT), fasting blood glucose (FBG), blood urea nitrogen (BUN) and Creatinine were tested in the national accredited laboratory in Shandong Provincial Qianfoshan Hospital following standard procedures.

### Definition of dyslipidemia

Dyslipidemia was defined according to 2016 Chinese guideline for the management of dyslipidemia proposed by guideline revision National Expert Committee [2]. Dyslipidemia was diagnosed as TG ≥ 2.3 mmol/L (≥200 mg/dl), and/ or LDL-C ≥ 4.1 mmol/L (≥160 mg/dl), and/ or TC ≥ 6.2 mmol/L (≥240 mg/dl), and/ or HDL-C ≤ 1.0 mmol/L (≤40 mg/dl) and/ or self-reported clinically diagnosed dyslipidemia.

### Statistical analyses

The baseline characteristics of the sample by group were compared using *t* tests for continuous variables, and *χ*^*2*^ tests for categorical variables. SPSS16.0 was used to run *t* tests and *χ*^*2*^ tests. A partial least squares path model (PLSPM) was employed to explore possible pathways from metabolic-related markers to dyslipidemia. The PLSPM is a variance-based structural equation modeling, which allows for the construction of a path model between a set of latent variables (inner model), each of which being represented by a block of correlated measured or manifest variables (outer model) [20].

In this study, latent variables including lipid metabolism factor (LMF), blood pressure factor (BPF), obesity condition factor (OCF), glucose metabolism factor (GMF), renal function factor (RFF), lifestyle factor and inflammatory factor (IF) were measured at baseline. The latent variable of dyslipidemia with manifest variables of LDL-C, TC, TG and HDL-C were measured at the end of follow-up. This representation indicated that LMF could manifest as TG, LDL-C, TC and HDL-C at baseline. BPF loaded on both SBP and DBP. OCF loaded on BMI. GMF loaded on FBG. Lifestyle factor loaded on smoking status and drinking status. RFF loaded on BUN and Creatinine. And IF loaded on GGT, WBC, NGC and MCC. The total effect (β) of each latent variable was the sum of direct and indirect effect on dyslipidemia. Lohmöller algorithm were used to estimate path coefficients and factor loadings [21]. Bootstrapping in PLS offers *t* test statistics that confirm statistical significance [21, 22]. The SmartPLS 2.0 was used to build PLSPM [23].

## Results

### Baseline characteristics

A total of 25,130 participants with a mean age of 42.4 ± 13.8 years were included in present study with 50,014 person-years of follow-up. Five thousand five hundred fifty-one participants developed dyslipidemia during 5-year follow-up. The total incidence density was 110.99/1000 person-years (5551/50014 person-years). Table 1 shows the baseline characteristics by the incident dyslipidemia status (dyslipidemia and non-dyslipidemia). Males, elders, participants with higher proportion of current smoking status and drinking status, BMI, TG, TC, LDL-C, SBP, DBP, GGT, NGC, MCC, WBC, FBG, BUN, Creatinine level and lower HDL-C level at baseline were likely to have new-onset dyslipidemia (Table 1).

**Table 1:** Baseline characteristics of participants grouped by dyslipidemia

Variables	Total	Dyslipidemia	Non-dyslipidemia	t/χ^2^ value	*P* value
Sex (males)	13,315 (53.0)	3855 (69.4)	9460 (48.3)	775.12	< 0.01
Age (years)	42.4 ± 13.8	45.4 ± 13.7	41.6 ± 13.7	−18.39	< 0.01
Smoking status				244.7	< 0.01
Never	18,806 (83.5)	3485 (75.9)	15,321 (85.4)		
Former	70 (0.3)	26 (0.6)	44 (0.2)		
Current	3654 (16.2)	1083 (23.5)	2571 (14.4)		
Drinking status				353.5	< 0.01
Never	15,905 (70.6)	2734 (59.5)	13,171 (73.4)		
Former	25 (0.1)	14 (0.3)	11 (0.1)		
Current	6600 (29.3)	1846 (40.2)	4754 (26.5)		
BMI (kg/m^2^)	23.5 ± 3.3	24.9 ± 3.1	23.1 ± 3.2	− 37.07	< 0.01
HDL-C (mmol/L)	1.6 ± 0.3	1.4 ± 0.3	1.6 ± 0.3	24.42	< 0.01
TG (mmol/L)	1.0 ± 0.3	1.2 ± 0.3	0.9 ± 0.3	− 60.39	< 0.01
TC (mmol/L)	4.6 ± 0.7	4.9 ± 0.7	4.5 ± 0.6	− 38.21	< 0.01
LDL-C (mmol/L)	2.6 ± 0.6	2.9 ± 0.5	2.5 ± 0.5	− 47.86	< 0.01
SBP (mmHg)	124.7 ± 17.5	129.2 ± 17.7	123.5 ± 17.2	− 21.73	< 0.01
DBP (mmHg)	78.2 ± 11.5	81.5 ± 11.4	77.3 ± 11.4	− 24.29	< 0.01
GGT (U/L)	21.9 ± 21.0	28.5 ± 29.7	20.1 ± 17.3	− 26.64	< 0.01
NGC (10^9^/L)	3.4 ± 1.1	3.5 ± 1.1	3.3 ± 1.1	− 9.1	< 0.01
MCC (10^9^/L)	0.3 ± 0.1	0.4 ± 0.1	0.3 ± 0.1	− 8.81	< 0.01
WBC (10^9^/L)	6.1 ± 1.4	6.3 ± 1.5	6.0 ± 1.4	− 12.74	< 0.01
FBG (mmol/L)	5.1 ± 0.5	5.2 ± 0.6	5.1 ± 0.5	−20.52	< 0.01
Creatinine (umol/L)	67.5 ± 17.2	70.4 ± 16.4	66.8 ± 17.3	−12.44	< 0.01
BUN (mmol/L)	4.8 ± 1.3	5.1 ± 1.2	4.7 ± 1.3	−17.5	< 0.01

*BMI* Body mass index, *HDL-C* high-density lipoprotein cholesterol, *TC* total cholesterol, *TG* triglyceride, *LDL-C* low-density lipoprotein cholesterol, *SBP* systolic blood pressure, *DBP* diastolic blood pressure, *GGT* γ-glutamyltransferase, *NGC* neutrophile granulocyte, *MCC* monocyte count, *WBC* white blood cell count, *FBG* fasting blood glucose, *BUN* blood urea nitrogen

### Pathway analysis using PLSPM

**Fig. 1:**
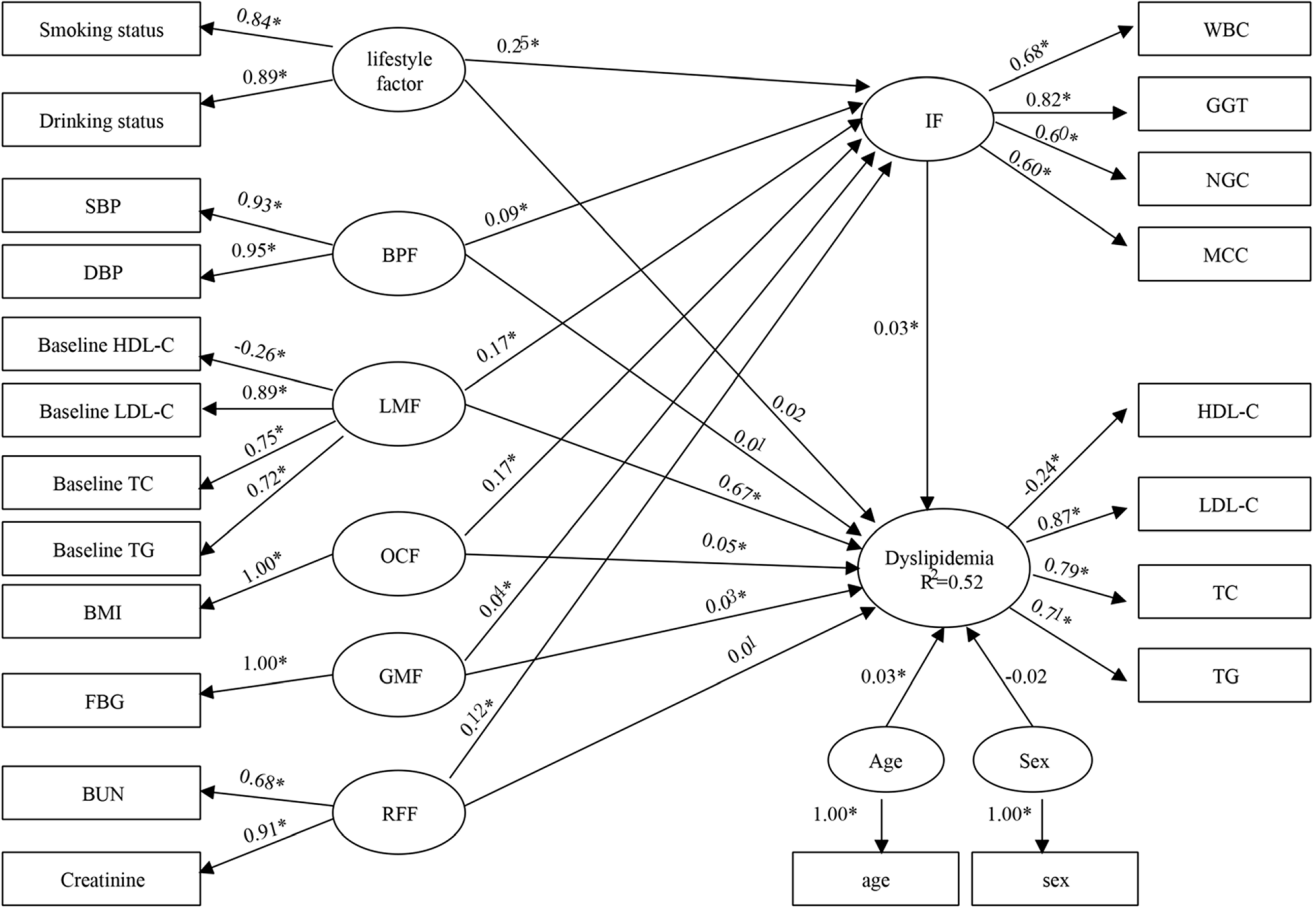
The partial least squares path model for metabolic-related markers and inflammatory factors associated with dyslipidemia. *P < 0.05

Figure 1 shows the results from the final PLSPM that illustrated possible pathways of metabolic-related biomarkers on dyslipidemia. The determination coefficient of the model (R^2^) was 0.52, indicating that seven latent variables explained 52% of the total variance and thus had an acceptable capacity in explanation of dyslipidemia.

In the outer model, the results showed that the manifest variable of baseline LDL-C was the most significant contributor in the formation of LMF (loading = 0.89), followed by baseline TC (loading = 0.75), baseline TG (loading = 0.72) and baseline HDL-C (loading = − 0.26). SBP and DBP had positive effects on the block of BPF with loading 0.93 and 0.95. BUN and Creatinine had positive effects on the block of RFF with loading 0.68 and 0.91. Smoking status and drinking status had positive effects on the block of lifestyle factor with loading 0.84 and 0.89. For the block of IF, GGT was the most important contributor (loading = 0.82), followed by WBC (loading = 0.68), NGC (loading = 0.60) and MCC (loading = 0.60).

For the inner model, LMF, OCF and GMF all had direct effect as well as indirect effect on dyslipidemia through IF. However, BPF, RFF and lifestyle factor only had indirect effect on dyslipidemia through IF. LMF was the most important risk factor [β = 0.68, including a direct effect of 0.67, and an indirect effect of 0.01 (0.17 × 0.03)] in the prediction of dyslipidemia, followed by OCF [β = 0.06, including a direct effect: 0.05, an indirect effect: 0.01 (0.17 × 0.03)] and GMF [β = 0.03, including a direct effect: 0.03, an indirect effect: 0.001 (0.04 × 0.03)] (Table 2).

**Table 2:** Bootstrapping test of path coefficients in the partial least square path model

Pathways	Path coefficient	t value
Age → dyslipidemia	0.03	6.71
Sex → dyslipidemia	−0.02	2.30
Lifestyle factor → dyslipidemia	0.02	3.61
GMF → dyslipidemia	0.03	6.31
OCF → dyslipidemia	0.05	8.13
LMF → dyslipidemia	0.67	138.37
BPF → dyslipidemia	0.01	1.97
RFF → dyslipidemia	0.01	1.04
IF → dyslipidemia	0.03	4.21
Lifestyle factor → IF	0.25	39.32
GMF → IF	0.04	4.42
OCF → IF	0.17	27.53
LMF → IF	0.17	25.16
BPF → IF	0.09	12.64
RFF → IF	0.12	17.30
Lifestyle factor → IF → dyslipidemia	0.25 × 0.03	–
GMF → IF → dyslipidemia	0.04 × 0.03	–
OCF → IF → dyslipidemia	0.17 × 0.03	–
LMF → IF → dyslipidemia	0.17 × 0.03	–
BPF → IF → dyslipidemia	0.09 × 0.03	–
RFF → IF → dyslipidemia	0.12 × 0.03	–

## Discussion

In the present study, we explored the effect of metabolic-related markers on dyslipidemia and the mediating role of inflammatory factor using reliable routine check-up data from a cohort study. We found that lipid metabolism factor, blood pressure factor, obesity condition factor, glucose metabolism factor, renal function factor and lifestyle factor had diverse impact on development of dyslipidemia, directly and (or) indirectly, and inflammatory factor played a mediating role in the associations. Meanwhile, modeling the data with PLSPM indicated that lipid levels at baseline conferred the most important dimensional variable in the prediction of dyslipidemia, followed by obesity.

Our results illustrated that lipid-related markers at baseline is the most significant contributor in the prediction of dyslipidemia, which is consistent with previous studies showing that the corresponding lipid and lipoprotein levels in early life were the strongest predictors for dyslipidemia [9, 24]. Similarly, there is ample evidence that adverse lipid levels in childhood could persist over time, and progress to adult dyslipidemia and other cardiovascular diseases [25, 26]. Furthermore, preventive efforts aimed at dyslipidemia that start in early life could delay progression to adverse outcomes. Findings from clinical trials conducted in individuals starting with lipid level lower than current guideline targets showed that the risk of major vascular events was significantly reduced by 21% for each 1-mmol/L (38.7-mg/dL) reduction in LDL-C [27]. Therefore, it is essential to monitor lipid levels and identify high-risk individuals as early as possible, so that targeted interventions can be taken to prevent development of dyslipidemia and its related complications.

In regard to obesity condition factor measured by BMI, the current study demonstrated a risk effect on dyslipidemia, similar to the findings from previous studies [7, 28]. Accumulating evidence indicate that a state of chronic inflammation play a crucial role in the pathogenesis of obesity-related metabolic dysfunction [29–31]. We found that obesity condition factor had direct effect as well as indirect effect on dyslipidemia through inflammation. Pathway analysis suggested that obesity had greater effect than other biomarkers on inflammation as indicated by the higher path coefficient. Experimental studies have confirmed as adipose tissue expands, there is greater infiltration of macrophage and lymphocyte, which lead to an increase in chronic inflammation [32, 33]. A epidemiological study suggested that the levels of inflammatory biomarkers increased with elevation of obesity degree and a strong association between the biomarkers and BMI was demonstrated [34]. A recent study suggested that elevated plasma levels of inflammatory markers were positively associated with increased risk of dyslipidemia [14]. Inflammatory cell infiltration within adipose tissue may be involved in altering adipocyte lipid and cytokine production, which may in turn have downstream effects on lipid metabolism disorder [33, 35, 36]. Furthermore, the present study showed that lifestyle factor has an indirect effect on dyslipidemia through inflammation. Therefore, the findings provide valuable insights for strategies of dyslipidemia prevention. Controlling weight and changing lifestyle could be an effective way to decrease the levels of inflammatory biomarkers, and prevent the development of dyslipidemia.

Hypertension and dyslipidemia are two of modifiable risk factors for cardiovascular disease, and coexistence of the two factors is often observed in clinical settings, which exerts more than an additive impact on the vascular endothelium, resulting in atherosclerosis [37, 38]. It is reported that hypertensive individuals more frequently display lipid abnormities than normotensive ones [39, 40]. Our finding confirmed that elevated levels of blood pressure were associated with inflammation, which important pathway lead to dyslipidemia. The vasoconstriction that characterized a chronic hypertensive state may involve in the association between hypertension and increased risk of dyslipidemia [41]. It has been found that this hemodynamic phenomenon may address an adverse effect on lipid metabolism. For example, vasoconstriction may slow down the disposal of various components of the lipid profile in adipose and hepatic tissue [42]. Therefore, our findings suggested that hypertension combined with inflammatory markers may serve as potential intervention targets for the management of dyslipidemia.

A major strength of our study is that data from a large-scale of routine health check-up population based on a longitudinal study could provide convincing support of the hypothesized relationship. Second, we used pathway analysis to explore the effect of metabolic markers simultaneously as a system of multiple pathways on dyslipidemia, and the mediating role of inflammation on the associations. Elucidating the underlying mechanisms could increase our understanding of the potential biological pathways to dyslipidemia and provide a novel perspective for the research of dyslipidemia. Third, applying PLSPM method to construct structural equation model (SEM) in our study have some advantages in methodological feature. Compared to covariance-based SEM, PLSPM is a “soft modeling” approach requiring few distributional assumptions, in which variables can be numerical, ordinal or nominal, and no need for normality assumptions [20]. However, several limitations in this study need consideration. First, some of biomarkers in the model may not be perfect markers of the relevant metabolic pathways, because of the particularity of data collecting from routine health check-up population. For example, in our study NGC, MCC, WBC and GGT, widely available biomarkers in routine health check-up, were used to reflect inflammation other than C reaction protein, nor tumor necrosis factor-α, interleukin-6. While the hematological parameters such as peripheral WBC count and GGT level may not be more robust, they have certain reference values for evaluating sub-clinical inflammation. Obesity factor was measured by BMI rather than waist circumference, which might be more sensitive indicator for dyslipidemia. Second, some potential confounders such as physical activity were not included in the model. These limitations may impose a modest constraint on the interpretation of these findings, but they should not substantively undermine the internal validity of the study.

## Conclusions

In conclusion, the findings from our study informed that future management and treatment of dyslipidemia should take comprehensive intervention strategies into consideration, which is of great practical significance to choose monitoring, prevention and intervention strategies in general population. Given that inflammation mediates the associations between metabolic markers and dyslipidemia, combined preventive strategies on metabolic biomarkers and inflammation may be more effective in reducing the risk of dyslipidemia.

## Data Availability

The datasets used in the current study are available from the corresponding author upon reasonable request.

## Abbreviations

BMI: Body mass index
BUN: Blood urea nitrogen
DBP: Diastolic blood pressure
FBG: Fasting blood glucose
GGT: γ-glutamyltransferase
GMF: Glucose metabolism factor
HDL-C: High-density lipoprotein cholesterol
IF: Inflammatory factor
LDL-C: Low-density lipoprotein cholesterol
LMF: Lipid metabolism factor
MCC: Monocyte count
NGC: Neutrophile granulocyte
OCF: Obesity condition factor
PLSPM: Partial least squares path model
RFF: Renal function factor
SBP: Systolic blood pressure
SEM: Structural equation model
TC: Total cholesterol
TG: Triglyceride
WBC: White blood cell count
